# A Hyperheuristic Approach for Location-Routing Problem of Cold Chain Logistics considering Fuel Consumption

**DOI:** 10.1155/2020/8395754

**Published:** 2020-01-04

**Authors:** Zheng Wang, Longlong Leng, Shun Wang, Gongfa Li, Yanwei Zhao

**Affiliations:** ^1^School of Computer Science and Technology, Zhejiang University of Technology, Hangzhou 310023, China; ^2^Key Laboratory of Special Equipment Manufacturing and Advanced Processing Technology, Ministry of Education, Zhejiang University of Technology, Hangzhou 310023, China; ^3^Key Laboratory of Metallurgical Equipment and Control Technology, Ministry of Education, Wuhan University of Science and Technology, Wuhan 430081, China

## Abstract

In response to violent market competition and demand for low-carbon economy, cold chain logistics companies have to pay attention to customer satisfaction and carbon emission for better development. In this paper, a biobjective mathematical model is established for cold chain logistics network in consideration of economic, social, and environmental benefits; in other words, the total cost and distribution period of cold chain logistics are optimized, while the total cost consists of cargo damage cost, refrigeration cost of refrigeration equipment, transportation cost, fuel consumption cost, penalty cost of time window, and operation cost of distribution centres. One multiobjective hyperheuristic optimization framework is proposed to address this multiobjective problem. In the framework, four selection strategies and four acceptance criteria for solution set are proposed to improve the performance of the multiobjective hyperheuristic framework. As known from a comparative study, the proposed algorithm had better overall performance than NSGA-II. Furthermore, instances of cold chain logistics are modelled and solved, and the resulting Pareto solution set offers diverse options for a decision maker to select an appropriate cold chain logistics distribution network in the interest of the logistics company.

## 1. Introduction

Cold chain logistics are developing rapidly with constant improvement of living standard of the people and increasing demand for fresh food [1]. In order to lower distribution cost of cold chain logistics effectively, some researchers began to study optimization of cold chain logistics distribution network [2]. However, when optimizing cold chain logistics distribution network, one has to consider economic benefit of logistics distribution, distribution-derived carbon emission and other environmental benefits, and timeliness of the logistics network.

In a practical cold chain logistics network, more than one objective may be considered in the system, including the minimum total cost, the smallest number of vehicles, the maximum customer satisfaction, the shortest travel path, and the least distribution time. Numerous researchers have studied multiobjective logistics optimization problem. With the shortest travel path and carbon dioxide emission as optimization objectives, Jemai et al. [3] established a vehicle routing problem (VRP) based on green logistics and solved it by NSGA-II optimization. Based on optimization objective functions of the minimum cost and the maximum customer satisfaction, Liu et al. [4] developed a mathematical model for multiobjective location-routing problem (LRP). Molina et al. [5] developed a heterogeneous fleet VRP model with the minimized cost and emission as objectives. Golmohammadi et al. [6] proposed a mathematical model for multiobjective LRPs with the minimum storage distribution cost and difference in vehicle-traveled distance as optimization objective functions. The above literatures are related to multiobjective logistics distribution network, but multiple performance metrics of logistics network are not taken into account: economic benefit, environmental benefit, and timeliness. Moreover, there are a dearth of literatures on LRP-based models for cold chain logistics. Wang et al. [1] proposed developing a single-objective model in consideration of carbon emission for cold chain logistics and solving it using hybrid genetic algorithm. However, logistics network timeliness has not yet been considered.

Hence, in this paper, properties of cold chain logistics are considered from the perspective of LRP-based model; not only economic and environmental benefits but also distribution timeliness are taken into account; thereby a biobjective LRP of cold chain logistics considering fuel consumption and distribution period is proposed, and one multiobjective hyperheuristic (MOHH) is proposed to model and solve this problem. Therefore, main contributions of this paper are described as follows:

- Problem model: with logistics cost and distribution period as objective functions, a multiobjective mathematical model for cold chain logistics is developed considering environmental benefit arising from fuel consumption and social benefits like customer satisfaction arising from customer time window.
- Solution algorithm: for the above model, MOHH is designed. Based on the framework nature of hyperheuristics, four selection strategies for low-level heuristics (LLHs) and four solution acceptance criteria are proposed.
- Management suggestions and comments: based on experimental results, several insights and suggestions about logistics distribution are provided from management perspective.

## 2. Methodology

### 2.1. Multiobjective Optimization Problem (MOP)

One typical MOP normally contains more than 2 conflicting or contradictory objectives and is also called multicriteria optimization problem [7]. Without loss of generality, one minimized MOP having *D*-dimensional decision variables and *m*-dimensional subobjectives can be expressed as

(1) $$\begin{array}{c} \begin{array}{cc} \underset{X\in \Omega }{\min}F\left(X\right)= & {\left(f_{1}\left(X\right),f_{2}\left(X\right),\ldots ,f_{m}\left(X\right)\right)}^{\text{T}}\\ \text{s}.\text{t}. & \left\{\begin{array}{c} g_{i}\left(X\right)\leq 0,i=1,2,\ldots ,q\\ h_{i}\left(X\right)=0,i=1,2,\ldots ,p, \end{array}\right. \end{array} \end{array}$$

where **X**=(*x*1, *x*2,…, *x* *D*) ∈ *R*^*D*^ is a *D*-dimensional decision vector and **F**(**X**) ∈ *R*^*m*^ is an *m*-dimensional objective vector, defining *m* mapping functions of decision space towards objective space; *R*^*D*^ and *R*^*m*^ are decision space and objective space, respectively; *g*_*i*_(**X**) ≤ 0(*i*=1,2,…, *q*) are *q* inequality constraints; and *h*_*i*_(**X**) = 0 (*i* = 1, 2,…, *p*) are *p* equality constraints.

On this basis, the following related definitions are given [7].

**Definition 1 (feasible solution).**

For one **X** ∈ *R*^*D*^, if two classes of constraints in equation (1) are satisfied, then **X** will be deemed as a feasible solution.

**Definition 2 (feasible solution set).**

A set of all feasible solutions is referred to as feasible solution set, that is, **X**_*f*_.

**Definition 3 (Pareto dominant).**

Suppose that **X**_*A*_ and **X**_*B*_ are two feasible solutions in the feasible solution set. When and only when the following constraints are satisfied,

(2) $$\begin{array}{c} \left\{\begin{array}{c} \forall i=1,2,\ldots ,m,f_{i}\left(X_{A}\right)\leq f_{i}\left(X_{B}\right),\\ \exists j=1,2,\ldots ,m,f_{j}\left(X_{A}\right)<f_{j}\left(X_{B}\right). \end{array}\right. \end{array}$$

where **X**_*A*_ dominate **X**_*B*_; in other words, compared with **X**_*B*_, **X**_*A*_ is a Pareto dominant, which can be expressed as **X**_*B*_ ≻ **X**_*A*_.

**Definition 4 (Pareto optimal solution).**

One solution **X**^*∗*^ ∈ **X**_*f*_ is referred to as Pareto optimal solution (or nondominated solution), when and only when the following condition is satisfied:

(3) $$\begin{array}{c} \exists X\in X_{f}:X≻X^{*}. \end{array}$$

**Definition 5 (Pareto optimal solution set).**

As a set of all Pareto optimal solutions, Pareto optimal solution set is defined as follows:

(4) $$\begin{array}{c} P^{*}≜\left\{X^{*}\left|\exists \right.X\in X_{f}:X≻X^{*}\right\}. \end{array}$$

**Definition 6 (Pareto front).**

A curved surface, composed of objective function values corresponding to all Pareto optimal solutions in Pareto optimal solution set *P*^*∗*^, is called Pareto front and denoted as *PF*^*∗*^:

(5) $$\begin{array}{c} PF^{*}≜\left\{F\left(X^{*}\right)={\left(f_{1}\left(X^{*}\right),f_{2}\left(X^{*}\right),\ldots ,f_{m}\left(X^{*}\right)\right)}^{\text{T}}|X^{*}\in P^{*}\right\}. \end{array}$$

### 2.2. Hyperheuristics

**Figure 1:**
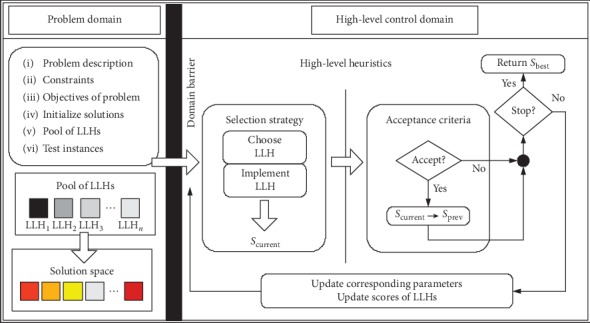
Selective hyperheuristic framework.

Metaheuristics (e.g., grey wolf optimization algorithm [8], nature-inspired optimization algorithm [9, 10], and quantum-behaved particle swarm optimization [11]) have been widely applied in optimization problems. However, to find solutions of bespoke nature in a new problem-based domain, it takes huge efforts and lengthy time to have a good command of effectiveness of domain operator combination [12]. Moreover, it is difficult to select the promising optimal parameters [13]. Although the machine learning methods have also been used to solve the above dilemmas, such as obstacle recognition based on machine learning [14], this paper focuses on the another strategy, namely, hyperheuristics, which are capable of solving the above problems effectively. Cowling et al. [15] defined “hyperheuristic” as “heuristics to choose heuristics” for the first time. Later, Burke et al. [16] expanded its definition: (i) heuristic selection and (ii) heuristic generation. Therefore, selection of optional LLHs is generally decided from indicators of spread or convergence for a candidate solution versus parent solution. As known from the above literature, an LLH can be defined as an operator (crossover or mutation operator) or a metaheuristic (e.g., NSGA-II and SPEA2). For a selective heuristic, there exists one universal simple framework, as shown in Figure 1.

The black zone is a domain barrier for isolating high-level heuristics (HLHs) from LLHs. The low-level problem domain contains data information of the real problem, LLHs used to search directly for problem space, and population information of the problem domain (chromosome, fitness, etc.). In high-level control domain, there are two strategies with different objectives: operator selection strategy and solution acceptance mechanism. The selection strategy is used to search for LLH-based space and monitor history information of LLH performance so as to select high-quality operators (for isolating any information related to the real problem), while the acceptance criteria judge whether parent solution *S*_p_ should be replaced depending on quality of children solution (or solution set) *S*_c_ or not and control search direction and convergence speed of the algorithm, where *S*_cu_ is the current solution. In addition, between HLHs and LLHs, there are information transmitters used to exchange and transmit information irrelevant to the problem domain, including choice information, acceptance strategy judgment information, improvement rate contributed by LLH, operator runtime and frequency, and number of consecutive improvement failures of current solution. Moreover, it is to highlight that any decision for high-level domain (selection or acceptance strategy) has to be isolated from real problem domain (such as encoding, crossover, and mutation methods); otherwise, hyperheuristic principles and objectives will be disrupting; in other words, generality and transplantability of the high-level control strategy are improved in order to apply to any problem domain.

Although there are plenty of multiobjective algorithms (such as NSGA-II, SPEA2, and multiobjective optimization based on an improved cross-entropy method [17]) for solving the practical problems in the real-world applications, this paper focuses on the MOHH, which has critical problems in the following aspects in its design:

1. Design of high-level selection strategy: performance of LLH may exhibit phased feature, that is, varying with search progress; for example, operator performance has inconsistent manifestations in the early and late phases of search. On this basis, researchers have designed different selection strategies depending on demand, such as choice function [18, 19] and sliding window-based FRR-MAB [20]
2. Approach to children solution evaluation: for a single-objective problem, it suffices to acquire fitness improvement rate (FIR) of children relative to parent. But, for an MOP, objective space is expanded to two or more dimensions, so FIR cannot be used directly to characterize an approach of replacing parent with children. Dominance relationship of children versus parent is utilized [20]. Two-stage sorting method is used to evaluate children solutions and reward corresponding LLHs [18, 19]
3. Solution acceptance mechanism: quality of solution acceptance mechanism has immediate impact on population diversification and intensification, thus influencing whether an algorithm is able to obtain satisfactory solutions. For single-objective problems, there are deterministic acceptance mechanisms such as All Moves (AM), Only Improving (OI), and noninferior solution acceptance and probabilistic (nondeterministic) acceptance mechanisms such as great deluge (GD), delay acceptance, simulated annealing (SA), and Monte Carlo
4. Domain barrier and information transmitter control: hyperheuristics are aimed at improving algorithm universality and transplantability so that the algorithm can be applied to real problems in different domains. So, there is crucial information in multiobjective problem domain: chromosome fitness; in other words, chromosome fitness can be used by high-level control strategy during multiobjective optimization likewise

Therefore, design methods are given in this paper for the above problems in MOHH design, respectively, so as to satisfy effectiveness and high performance of solving a biobjective LRP of cold chain logistics considering fuel consumption and distribution period.

### 2.3. Mathematical Model of Biobjective LRP of Cold Chain Logistics considering Fuel Consumption and Distribution Period

#### 2.3.1. Problem Formulation, Assumptions, and Definitions of Variables

LRP of cold chain logistics studied in this paper is a distribution centre-to-customer supply chain. Suppose that the cold chain logistics network is a directed network composed of distribution centres and customer points; that is, *N* = {*N*_C_, *N*_D_} represents a set of all points on the logistics network, where *N*_C_ represents a set of customer points; it is a weighted directed network where point-to-point distances are the weights, including the distance between a distribution centre and a customer and the distance between customers. Optimization of a logistics network consists of two major aspects: reasonable distribution centre selection and vehicle routing. To study instances of cold chain logistics network reasonably, the following assumptions are made:

*(1) Urban Traffic Status Is Relatively Stable During Distribution Period*. Changes in urban road traffic status are not chaotic; in general, roads will be congested during morning and evening peak hours in a day if no accident occurs; congestion status lasts for about two hours, while during other hours, roads will not be so congested, and road traffic does not vary much.

*(2) The Distribution Vehicle Remains in Uniform Speed State*. Suppose that urban road congestion status is constant; it is very difficult to predict vehicle speed in LRP and VRP, as the speed varies somewhat with time interval, and it is very hard to describe the speed variation model with mathematical functions; therefore, in this study of LRP of cold chain logistics, speed of the distribution vehicle is deemed uniform.

*(3) Other Assumptive Conditions*. Each distribution centre is equipped with vehicles; a distribution vehicle transports cold chain logistics products from each distribution centre to various customers and returns to the original distribution centre. The maximum load weight of each vehicle is *Q*, no demand of one customer exceeds the maximum load weight of each vehicle, demand of one customer can be satisfied by one vehicle only through distribution service, and one vehicle is able to serve many times. If drivers have the same driving habit and driving skill and each distribution centre has abundant inventory, then cargo shortage will not occur.

Notations and variables of the model are defined as follows: candidate distribution centre set *N*_D_ = {1, 2,…, *m*}, customer set *N*_C_ = {1, 2,…, *n*}, available vehicle set *K* = {1, 2,…, *φ*}, all-point set *N*=*N*_D_ ∪ *N*_C_, and edge set *E* = {(*i*, *j*): *i* ∈ *V*, *j* ∈ *V*, *i* ≠ *j*}\{(*i*, *j*): *i* ∈ *J*, *j* ∈ *J*, *i* ≠ *j*}; transportation distance corresponding to each edge is *d*_*ij*_ and travel speed is *v*_*ij*_; customer *i* ∈ *N*_C_ has nonnegative cargo allocation demand *D*_*i*_ and time window requirement (ET_*i*_, LT_*i*_), while *w*_*i*_ is customer service time; vehicle type is the same and the maximum capacity is CV; distribution centre *i* ∈ *N*_D_ has an enabling cost of *O*_*i*_ and the maximum capacity of CD_*i*_.

The following are decision variables: *x*_*ijk*_ is 1 if edge (*i*, *j*) serves vehicle *k*; otherwise *x*_*ijk*_ is 0; *y*_*j*_ is 1 when distribution centre *j* is enabled; otherwise *y*_*j*_ is 0; *z*_*ij*_ is 1 when customer *i* is served by distribution centre *j*; otherwise *z*_*ij*_ is 0.

The following are additional variables: *L*_*ijk*_ represents dynamic load of vehicle *k* on edge (*i*, *j*); AT_*ik*_ is the time point at which vehicle *k* arrives at node *i*.

Therefore, based on the above model assumptions and definitions of notations and variables, a multiobjective mathematical model can be given in this paper.

#### 2.3.2. Model Composition

The biobjective model in this paper is designed to optimize total logistics cost and distribution period; the total logistics cost consists of fuel consumption cost, cargo damage cost, transportation cost, refrigeration cost of refrigerated trucks, penalty cost of time window, and operation cost of distribution centres, as detailed below.

*(1) Fuel Consumption Cost*. According to a report on the website of the government of Japan [21], fuel consumption of one type of vehicles is directly proportional to load weight. In light of this, Xiao et al. [22] assumed that a vehicle runs at a constant speed, only considered impacts of load weight and traveled distance on vehicle fuel emission, and split vehicle weight into dead weight *M*_0_ and load weight *M*_1_; then Fuel Consumption Rate (FCR) can be calculated using the following formula:

(6) $$\begin{array}{c} \rho \left(M_{1}\right)=\alpha \left(M_{0}+M_{1}\right)+b. \end{array}$$

The maximum load weight that can be borne by a vehicle is defined as CV; then, FCRs at zero load and full load can be expressed as *ρ*_0_ = *αM*_0_ + *b* and *ρ*^*∗*^ = *α*(*M*_0_ + CV) + *b*, respectively. Thus, FCR per unit distance is

(7) $$\begin{array}{c} \rho \left(M_{1}\right)=\rho_{0}+\left(\rho^{*}-\rho_{0}\right)\times \frac{M_{1}}{\text{CV}}. \end{array}$$

Therefore, the fuel consumption generated by vehicle *k* under load *L*_*ijk*_ is

(8) $$\begin{array}{c} {\text{FC}}_{ijk}=\left(\rho_{0}+\left(\rho^{*}-\rho_{0}\right)\times \frac{L_{ijk}}{\text{CV}}\right)\times d_{ij}x_{ijk}, \end{array}$$

and, for this problem mentioned, fuel consumption cost is

(9) $$\begin{array}{c} C_{1}=c_{0}\underset{i\in N}{\sum }\underset{j\in N}{\sum }\underset{k\in K}{\sum }{\text{FC}}_{ijk}, \end{array}$$

where *c*_0_ represents unit fuel cost (Yuan/L).

*(2) Cargo Damage Cost of Cold Chain Logistics*. Based on the rationale of previous studies on cold chain logistics products, researchers incorporated cargo damage cost analysis into distribution routing optimization of cold chain logistics network. To facilitate the study, suppose that cold chain logistics products in the whole cold chain logistics distribution network are stored at a constant temperature in stable environment. The top consideration is quality degradation of cold chain logistics products over time, and according to food science principles combined with deterioration rate function of cold chain logistics products, Qi [23] induced a mathematical relationship between product quality change ∆*Q* and original quality *Q*_0_ as follows:

(10) $$\begin{array}{c} lqm=\frac{\Delta Q}{Q_{0}}\times 100\%=\left\{1-e^{\sum_{i\in N}\sum_{j\in N}\left(-S\cdot t_{ij}e^{-\left(E_{a}/RT\right)}\right)}\right\}\times 100\%, \end{array}$$

where *t*_*ij*_ = *d*_*ij*_/*v*_*ij*_. Cargo damage of cold chain logistics is related to logistics distribution time only, and cargo damage cost is expressed as follows:

(11) $$\begin{array}{c} C_{2}=\left\{\underset{i\in N}{\sum }\underset{j\in N}{\sum }\underset{k\in K}{\sum }L_{ijk}\left[1-e^{-\varphi \left(d_{ij}/v_{ij}\right)}\right]\right\}\times 100\%. \end{array}$$

Let *φ* be time sensitivity coefficient; its expression is

(12) $$\begin{array}{c} \varphi =S\cdot e^{-\left(E_{a}/RT\right)}. \end{array}$$

*(3) Refrigeration Cost of Refrigeration Equipment*. In the course of distribution in cold chain logistics, a refrigeration equipment is not fully closed; its external factors such as solar radiation will result in a temperature gradient between inner and outer surfaces so as to increase thermal load inside the refrigeration equipment. The evaporator in the refrigeration equipment will refrigerate the cargo until overall thermal load is balanced, and eventually, constant temperature is achieved. Fu [24] performed thermal equilibrium analysis by thermal equilibrium method frequently used in energy consumption analysis of building system, and in cold chain logistics distribution, additional heat of refrigeration equipment arises from such factors as heat exchange between internal and external walls of refrigeration equipment, heat diffusion of products and equipment in cold chain logistics, and hot air soaking. For the brevity of study, only heat load *H*_1_ due to high-ratio heat exchange between internal and external walls of refrigeration equipment and heat load *H*_2_ due to high-ratio heat exchange upon door opening are considered. *H*_1_ is expressed as

(13) $$\begin{array}{c} H_{1}=R_{1}S\left(T-T_{0}\right), \end{array}$$

where *R*_1_ is the thermal conductivity of refrigeration equipment in W/(m^2^·K); *S* is the heat transfer area in m^2^ of refrigeration equipment on a refrigerated truck; *T* is the temperature outside refrigeration equipment in K; and *T*_0_ is the product storage temperature of cold chain logistics in K. *H*_2_ is expressed as follows:

(14) $$\begin{array}{c} H_{2}=R_{2}S_{\text{d}}\left(T-T_{0}\right), \end{array}$$

where *R*_2_ is the thermal conductivity of air in W/(m^2^·K), *S*_d_ represents the door area of refrigeration equipment in m^2^, and *w*_2_ is a parameter of unit refrigeration cost; the refrigeration cost during cold chain logistics network distribution can be expressed as

(15) $$\begin{array}{c} C_{4}=w_{2}\left(H_{1}\underset{i\in N}{\sum }\underset{j\in N}{\sum }\underset{k\in K}{\sum }\frac{d_{ij}}{v_{ij}}x_{ijk}+H_{2}\underset{i\in N}{\sum }\underset{k\in K}{\sum }w_{i}y_{ik}\right). \end{array}$$

*(4) Transportation Cost*. When cold chain logistics products are distributed from a distribution centre to a customer, transportation cost will be generated, including management cost of distribution vehicle drivers and transportation staff, toll, and vehicle loss cost, while unit transportation cost per vehicle is assumed to be known and fixed in the logistics system. Transportation cost of all vehicles is related to transportation distance and calculated using the following formula:

(16) $$\begin{array}{c} C_{3}=\varphi \underset{i\in N}{\sum }\underset{j\in N}{\sum }\underset{k\in K}{\sum }x_{ijk}d_{ij}, \end{array}$$

where *φ* is the cost per traveling distance.

*(5) Penalty Cost of Time Window*. As products in cold chain logistics are perishable, the customer demands that the logistics company distribute cold chain logistics products in a specified time frame. To improve logistics service level and customer satisfaction, penalty cost of time window is incorporated into cold chain logistics distribution routing objective function as follows:

(17) $$\begin{array}{c} P_{k}\left(i\right)=\left\{\begin{array}{cc} \alpha \left({\text{ET}}_{i}-{\text{AT}}_{ik}\right), & {\text{AT}}_{ik}<{\text{ET}}_{i},\\ 0, & {\text{ET}}_{i}\leq {\text{AT}}_{ik}\leq {\text{LT}}_{i},\\ \beta \left({\text{AT}}_{ik}-{\text{LT}}_{i}\right), & {\text{LT}}_{i}<{\text{AT}}_{ik}. \end{array}\right. \end{array}$$

where *P*_*k*_(*i*) represents penalty cost of time window that may be paid when vehicle *k* is serving customer *i*; AT_*ik*_ represents the time point at which vehicle *k* arrives at customer point *i*; *α* is a penalty coefficient for the case that a distribution vehicle arrives at a customer point at a time earlier than the start time of time window; *β* is a penalty coefficient for the case that a distribution vehicle arrives at a customer point at a time later than the start time of time window; ET_*i*_ and LT_*i*_ represent the start time and end time of time window, respectively. Therefore, penalty cost of time window can be expressed as

(18) $$\begin{array}{c} C_{5}=\underset{i\in N_{C}}{\sum }\underset{k\in K}{\sum }P_{k}\left(i\right). \end{array}$$

*(6) Operation Cost of Distribution Centres*. To ensure normal operation of cold chain logistics network, labour cost, equipment maintenance cost, and utilities have to be invested; the above costs altogether constitute operation cost of distribution centres in cold chain logistics. It is assumed that daily investment is basically unchanged in practical operation; thus distribution centres and operation costs are assumed to be fixed known conditions. Hence operation cost of distribution centres can be expressed as

(19) $$\begin{array}{c} C_{6}=\underset{i\in N_{D}}{\sum }O_{i}Y_{i}. \end{array}$$

#### 2.3.3. Location-Routing Optimization Modelling for Cold Chain Logistics

Based on the above model assumptions and definitions of notations and variables, a mathematical model was developed as follows:

(20) $$\begin{array}{c} \text{min Cost}=C_{1}+C_{2}+C_{3}+C_{4}+C_{5}+C_{6}, \end{array}$$

(21) $$\begin{array}{c} \min TT=\underset{i\in K}{\max}\left\{T_{i}\right\}, \end{array}$$

(22) $$\begin{array}{c} \underset{i\in N}{\sum }x_{ijk}=1,\;\forall j\in N_{\text{C}},k\in K, \end{array}$$

(23) $$\begin{array}{c} \underset{i\in N}{\sum }x_{ihk}-\underset{j\in N}{\sum }x_{hjk}=0,\;\forall h\in N_{\text{C}},k\in K, \end{array}$$

(24) $$\begin{array}{c} \underset{j\in N_{\text{D}}}{\sum }z_{ij}=1,\;\forall i\in N_{\text{C}}, \end{array}$$

(25) $$\begin{array}{c} z_{ij}\leq Y_{j},\;\forall i\in N_{\text{C}},j\in N_{\text{D}}, \end{array}$$

(26) $$\begin{array}{c} \underset{i\in N_{\text{C}}}{\sum }x_{ik}\leq Y_{k},\;\forall k\in N_{\text{D}}, \end{array}$$

(27) $$\begin{array}{c} x_{ink}+z_{ij}+\underset{m\in N_{\text{D}},m\neq j}{\sum }z_{nm}\leq 2,\;\forall i,n\in N_{\text{C}},i\neq n,j\in N_{\text{D}}, \end{array}$$

(28) $$\begin{array}{c} \underset{i\in N_{\text{C}}}{\sum }\underset{k\in N_{\text{D}}}{\sum }D_{i}z_{ik}\leq {\text{CD}}_{k}Y_{k},\;\forall k\in N_{\text{D}}, \end{array}$$

(29) $$\begin{array}{c} \underset{i\in N_{\text{C}}}{\sum }\underset{j\in N}{\sum }D_{i}x_{ijk}\leq \text{CV}, \end{array}$$

(30) $$\begin{array}{c} {\text{AT}}_{ik}={\text{AT}}_{jk}+t_{ij}+w_{jk},\;\forall i,j\in N_{\text{C}}, \end{array}$$

(31) $$\begin{array}{c} {\text{ET}}_{i}\leq {\text{AT}}_{ik}\leq {\text{LT}}_{i},\;\forall i\in N_{\text{C}},k\in K. \end{array}$$

Equations (20) and (21) are objective functions. Objective (20) represents minimization of total cost during optimization of cold chain logistics LRP, including fuel consumption cost, cargo damage cost, transportation cost, refrigeration cost of refrigerated truck, penalty cost of time window, and operation cost of distribution centre; objective (21) represents the shortest vehicle distribution time. Constraint (22) ensures that each customer is served once; constraint (23) ensures that the vehicle has to leave after serving a customer; constraint (24) indicates that each customer can be assigned to one distribution centre only; constraints (25)–(27) indicate that the enabled distribution centre has to serve a customer; constraint (28) ensures that total demand of customers served by each distribution centre is not more than capacity of the distribution centre; constraint (29) ensures that total volume of cargoes carried by each vehicle is not more than its loading capacity; constraint (30) represents temporal connection between two consecutive customer points in a vehicle distribution path; and constraint (31) indicates that the time when a vehicle arrives at a customer point may not be out of the permissible time interval.

### 2.4. MOHH

Conventional methods for solving MOP include weighting method, constraint method, and hybrid method. In recent years, Multiobjective Evolutionary Algorithms (MOEAs), as one kind of new methods for solving MOP, have been developed gradually. The most frequently used MOEAs are Genetic Algorithms (GAs), or multiobjective GAs, and representative GAs include VEGA, HLGA, MOGA, NPGA, and NSGA [25]. In this paper, MOHH is proposed for solving cold chain logistics-based multiobjective LRP model and distribution routing for cold chain logistics and is compared with Nondominated Sorting Genetic Algorithm II (NSGA-II) often used to solve MOP using elitist strategy [26]. NSGA-II employs simple yet efficient nondominated sorting mechanism so as to capture Pareto front and Pareto solution set with good distribution.

Problem domain and algorithm domain are designed below. The problem domain involves chromosome encoding and LLHs for optimization, while in high-level domain, four high-level selection strategies and acceptance mechanisms are designed; and finally, a multiobjective hyperheuristic framework is given.

#### 2.4.1. Problem Domain

*(1) Chromosome Encoding*. Direct encoding is used. If there are three distribution centres and 10 customers, 0 represents the distribution centre, and 1–10 represent customer numbers; then a distribution centre can be represented by 11–13. Customer sequence is generated randomly, for example, 9-5-3-2-4-1-6-8-7-10, and sequence location order represents the order of a vehicle arriving at a customer. Thereafter, according to principles “customer demand served by a vehicle may not exceed load weight of the vehicle” and “customer demand served by a distribution centre may not exceed capacity of the distribution centre,” distribution centres are allocated by centroid method; hence initialization path is converted to the following: 12-9-5-3-12; 11-2-4-1-6-11; 13-8-7-10-13. The first path represents that a vehicle departs from distribution centre no. 2 and distributes goods to customers nos. 9, 5, and 3 and then returns to the original distribution centre.

*(2) LLHs*. LLHs can be classified into mutation heuristic, ruin-recreate heuristic, local search heuristic, and crossover heuristic. LLHs are used to manipulate solution space directly as follows:

(i) Mutation heuristic:
    LLH_1_: 2-opt: select arbitrarily one path from parent solution and swap locations of adjacent customer points so as to generate a new children solution
    LLH_2_: Or-opt: select arbitrarily one path from parent solution and insert two adjacent customer points into other locations so as to generate a new children solution
    LLH_3_: interchange: select randomly two paths from parent solution and swap locations of any two customer points so as to generate a new children solution
    LLH_4_: replace: select randomly one path and swap locations of any two customer points
    LLH_5_: shift: select randomly one path from parent solution while enabling a new distribution centre for this path so as to generate a new children solution
    LLH_6_: interchange: select randomly two paths from parent solution and swap their distribution centres so as to generate a new children solution
(ii) Ruin-recreate heuristic:
    LLH_7_: location-based radial ruin: select any one customer point as “reference customer,” and remove other customers at a probability of 1%–10% according to a rule of approaching “reference customer” in location, so as to generate a new children solution
(iii) Local search heuristic:
    LLH_8_: interchange: as in the case of LLH_4_, but this operator returns the improved children solution only
    LLH_9_: shift: the operation is basically the same as that of LLH_3_, except that LLH_9_ selects a customer point according to “the best improvement” criterion, inserts the customer point into random location of the original path or any other path, and returns an improved children solution only
    LLH_10_: 2-opt^*∗*^: select randomly two paths from parent solution, choose one location according to “the best improvement” criterion, swap all customer points behind the location, and return an improved solution only
    LLH_11_: GENI: calculate the distance between any two customers on different paths in parent solution, select the shortest distance as reference distance, remove customer points nearest to the reference distance, rearrange these customers into a new path, and return an improved children solution only
(iv) Crossover heuristic:
    LLH_12_: combine: select randomly two parent paths, copy one of the parent paths at a probability of 25%–75% to generate a subpath, add paths that originate from another parent path and are not contradictory against this subpath, and eventually arrange arbitrarily the remaining customer points
    LLH_13_: longest combine: select randomly two parent paths, sort all paths in descending order of number of customer points served, add all paths without repeated customers to generate a subpath, and eventually arrange arbitrarily the remaining customer points

#### 2.4.2. High-Level Selection Strategies

*(1) Simple Random (SR)*. SR selects randomly LLHs in each iteration and is usually used as a reference for comparison with any other strategy.

*(2) Tabu Search (TS)*. TS is to prohibit repeating previous operation. To address a defect that local neighbourhood search algorithm gets readily trapped in local optimal points, TS algorithm formulates a tabu list to record searched local optimal points and not to search or search selectively elements in the tabu list in next iteration; thereby trap in local optimum is eliminated and global optimization objective is achieved. TS algorithm is an extension of local neighbourhood search and a heuristic for global neighbourhood search and gradual optimization.

As an HLH strategy for MOHH, TS scores performance of each LLH and thereby selects an operator for current iteration and updates scores using Reinforcement Learning mechanism; in other words, if a children solution arising from manipulation of the LLH improves parent solution, then score of this LLH will be added; otherwise it will be deducted.

*(3) Choice Function (CF)*. CF selects an LLH based on three different metrics; the first metric records previous performance of each LLH, denoted as *f*_1_, and LLH performance of each LLH can be expressed as

(32) $$\begin{array}{c} f_{1}=\underset{n}{\sum }\eta^{n-1}\frac{I_{n}\left({\text{LLH}}_{j}\right)}{T_{n}\left({\text{LLH}}_{j}\right)}, \end{array}$$

where *I*_*n*_(LLH_*j*_) and *T*_*n*_(LLH_*j*_) are the improvement value of each previously called heuristic and time to call the heuristic, respectively, and *η* is a coefficient ranging from 0 to 1.

The second metric attempts to capture connection between LLHs and can be expressed using the following formula:

(33) $$\begin{array}{c} f_{2}\left({\text{LLH}}_{k},{\text{LLH}}_{j}\right)=\underset{n}{\sum }\mu^{n-1}\frac{I_{n}\left({\text{LLH}}_{k},{\text{LLH}}_{j}\right)}{T_{n}\left({\text{LLH}}_{k},{\text{LLH}}_{j}\right)}, \end{array}$$

where *I*_*n*_(LLH_*k*_, LLH_*j*_) and *T*_*n*_(LLH_*k*_, LLH_*j*_) represent FIR of LLH_*j*_ following LLH_*k*_ and call time of LLH_*j*_ following LLH_*k*_, respectively. Likewise, *μ* is a coefficient ranging from 0 to 1.

The third metric is the time period since CF selects the last LLH_*j*_:

(34) $$\begin{array}{c} f_{3}\left({\text{LLH}}_{j}\right)=\tau \left({\text{LLH}}_{j}\right). \end{array}$$

To rank LLHs, a score is given to each LLH:

(35) $$\begin{array}{c} F\left({\text{LLH}}_{j}\right)=e\times f_{1}\left({\text{LLH}}_{j}\right)+f\times f_{2}\left({\text{LLH}}_{j}\right)+g\times f_{3}\left({\text{LLH}}_{j}\right), \end{array}$$

where *e*, *f*, and *g* represent weights of three metrics, respectively.

*(4) Ant-Inspired Selection (AS)* [27]. Ant colony optimization algorithm is a native heuristic of ant behaviour or ant population behaviour, used to solve hard combinatorial optimization problem. Ant colony algorithm applied in hyperheuristics is able to provide an HLH strategy so as to construct good LLH sequence. Each ant determines the next fixed point based on the pheromone in each path (each vertex represents an LLH), and once the ant arrives at a new vertex, it will apply the LLH corresponding to the vertex. Unlike the ant colony algorithm in combinatorial optimization problem solving mode, an ant colony manipulates LLHs; thus each ant is permitted to visit one point with multiple times, indicating that each heuristic may operate multiple times in one iteration. Details of the design are described in literature [27].

#### 2.4.3. High-Level Acceptance Mechanisms

Acceptance criteria are used to judge whether a children solution can replace parent solution or not, and its performance has immediate impact on convergence speed and optimization accuracy of a hyperheuristic [28]. It is therefore very important to determine superiority or inferiority of a children solution to parent solution in design of acceptance criteria. The following strategy is used in this paper: in a randomly selected objective function, as long as a children solution has a smaller fitness than parent solution, this children solution will be regarded to be superior to the parent solution and can go to the next iteration in place of the parent solution. This strategy is also used in the above three selection strategies.

1. All moves (AM): all solutions are accepted regardless of improvement
2. Simulated annealing (SA): it is similar to SA algorithm. Set initial temperature *T* and cooling coefficient, and update annealing temperature *T* = *T* × *λ* after each iteration. Apply an LLH to each iteration, accept an improved solution if any, and accept an unimproved solution at a certain probability expressed as *p* = *e*^∆/*T*^, where ∆ represents the change of objective function
3. Great deluge (GD) [28]: set a lower bound (LB) which is an optimal solution in current iteration. Apply an LLH to each iteration, accept an improved solution if any, and accept an unimproved solution in presence of the following case: *f*_temp_ < LB + (*f*_0_ − LB) × (1 − iter*/T*_max_), where *f*_temp_ represents a children solution, *f*_0_ represents initial solution, iter represents number of current iterations, and *T*_max_ represents the maximum number of iterations
4. Only improving (OI): accept improved solution only and eliminate all inferior solutions

#### 2.4.4. MOHH Framework

In a LRP model, the method focuses on designing distribution centres and customer groups, and the first job is to address distribution path between customers. The process is detailed as follows:

Step 1: initialize the population by encoding customers directly: suppose each chromosome has 10 customer points and 3 distribution centres, 1–10 represent customer numbers, and 0 represents a distribution centre. Distribution centres can be represented by 0(1)-0(3), respectively.
Step 2: initialize parameters of an HLH strategy.
Step 3: optimize population *P*:
    Step 3.1: select one of two objectives (*f*_1_, *f*_2_) at an equal probability randomly.
    Step 3.2: select an LLH based on its performance.
    Step 3.3: calculate the value of the selected objective function *f*_*n*_.
    Step 3.4: calculate the value of another objective function.
    Step 3.5: update performance metrics of the LLH.
Step 4: update Pareto solution set by nondominated sorting.
Step 5: evaluate a children solution according to an acceptance mechanism in acceptance criteria and opt to accept the children solution or not.
Step 6: update parameters related to HLH strategy and LLH score.
Step 7: judge whether termination condition is satisfied or not; if yes, then stop iteration and output optimal solution; otherwise, return to step 3.
Step 8: generate Pareto front.

## 3. Simulation Results and Analysis

### 3.1. Parameter Configurations

The MOHH was coded in parallel in MATLAB 2015b using a 2.60 GHz Intel Core i5-3230K with 4 GB of RAM and running Windows 10.

The parameter configurations were set as follows. The size of population was set to 100; the maximum number of iterations was set to 5000. In the choice function, three weights were 0.5, 0.2, and 0.3. In the ant-inspired selection, we followed the default values proposed by Wang et al. [27]: length of route LP = 11; number of ants *m* = 15; factors *ε* = 0.001 and *σ* = 1.001.

### 3.2. Performance Metrics

To validate performance of the proposed MOHH algorithm, the following three metrics were used:

1. Number of Pareto solutions (NPS): this metric is used to determine the number of Pareto solutions obtained by the algorithm.
2. Spacing metric (SM): this metric is used to determine distribution uniformity of Pareto solution set:

(36) $$\begin{array}{c} S={\left[\frac{1}{n}\overset{n}{\underset{i=1}{\sum }}{\left(\bar{d}-d_{i}\right)}^{2}\right]}^{1/2}, \end{array}$$

  where *d*_*i*_ represents the Euclidean distance from Pareto solution *i* to the nearest point in real Pareto solution set and $\bar{d}$ represents mean of all *d*_*i*_ values. The real Pareto solution set refers to nondominated solution set consisting of all Pareto solutions.
(3) Diversification metric (DM): this metric is used to measure diversity of Pareto solution set:

(37) $$\begin{array}{c} D=\sqrt{\overset{n}{\underset{i=1}{\sum }}\max\left\|x_{t}^{i}-y_{t}^{i}\right\|}, \end{array}$$

  where ‖*x*_*t*_^*i*^ − *y*_*t*_^*i*^‖ represents the Euclidean distance between nondominated solutions *x*_*t*_^*i*^ and *y*_*t*_^*i*^.

### 3.3. Experimental Comparison of HLH Strategies

To obtain better results in solving a biobjective LRP of cold chain logistics, MOHH algorithm was optimized by HLH strategy first; in other words, the best combination of selection strategy and acceptance criteria was found by comparing experimental results. One of Solomon reference instances was chosen for model solving, and as a standard LRP problem has neither refrigeration cost nor cargo damage cost, the total cost calculated in this section excludes refrigeration cost and cargo damage cost. Model solving results of HLH strategies are shown in Table 1.

Statistics NPS, SM, and DM are compared in Table 2. As can be found in Tables 1 and 2, AS-GD and TS-SA gave rise to more Pareto solutions than other combinations of HLH strategies; HLH strategy combination TS-SA resulted in smaller SM metric of nondominated solution set and smaller spacing, indicating that HLH strategy TS-SA results in more uniform distribution of nondominated solution set, as compared with other HLH strategies; in comparison, TS-GD, TS-OI, TS-SA, and AS-GD metrics had better DM values than other HLH strategy combinations.

**Figure 2:**
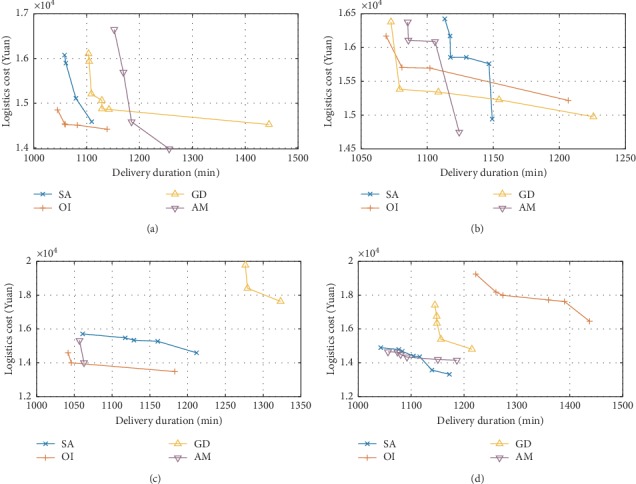
Profiles of Pareto solution set in four selection strategies. Pareto front under (a) AS, (b) CF, (c) SR, and (d) TS.

Figure 2 shows Pareto fronts obtained when selection strategies AS, CF, SR, and TS are combined with four different acceptance criteria, respectively. As can be found in the figure, when TS acts as a selection strategy, more Pareto solutions were obtained in more uniform distribution, and when SA acts as acceptance criteria, solution metrics NPS, SM, and DM were superior to other acceptance criteria.

In summary, when MOHH was applied to biobjective LRP optimization, the Pareto solution set obtained subject to selection strategy TS and acceptance criteria SA gave rise to good values of various evaluation metrics; thus TS-SA as a HLH strategy has better effect of solving biobjective LRPs.

### 3.4. MOHH Validation Analysis

#### 3.4.1. Statistical Analysis of LLH Utilization

**Figure 3:**
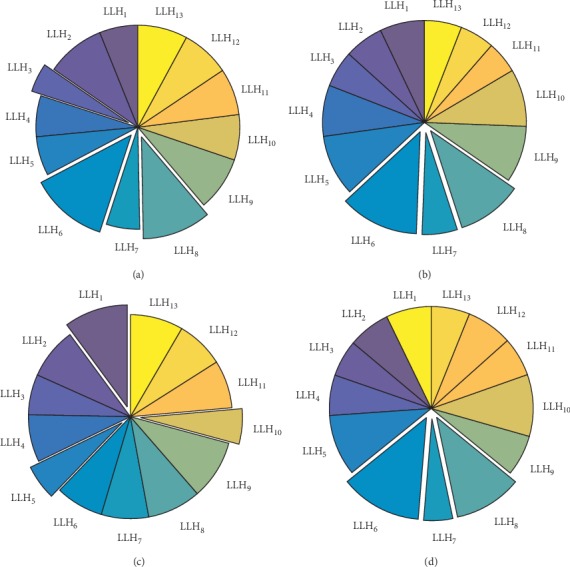
Mean operator utilization data of four selection strategies. Usage rate of operators under (a) AS, (b) CF, (c) SR, and (d) TS.

Two new LLHs were added to improve solving biobjective problems by optimization, utilization data and acceptance rates of LLHs were statistically analysed during algorithm optimization, and performances of HLH strategies and LLHs in optimization were analysed in order to study the impact of MOHH algorithm framework on multiobjective optimization.  Figure 3 shows the statistical results of LLH utilization.

Figure 3 shows that, among all LLHs, LLH_6_ had the highest utilization with a mean utilization of 11.3%; utilization of LLH_8_ reached high values, too, with a mean utilization of 10.2%, whereas mean utilization of LLH_7_ was merely 5.8%; except that LLHs were randomly selected in SR selection strategy, LLHs were selected by probability in all the remaining selection strategies, and the probability was determined based on performance of an LLH during algorithm iteration; it can therefore be inferred that LLH_6_ and LLH_8_ have better performance than other LLHs. In utilization statistics of LLH_6_, selection strategy TS showed the highest utilization of LLH_6_; thus it can be inferred that TS enables better performance of LLH_6_, a LLH_7_ utilization of just 4.6%, and lower utilization of worse-performance LLHs; therefore, in solving biobjective LRP, selection strategy TS is superior to three other selection strategies, which also validates experimental conclusions in this paper.

#### 3.4.2. Statistical Analysis of Acceptance Rates in LLHs

**Figure 4:**
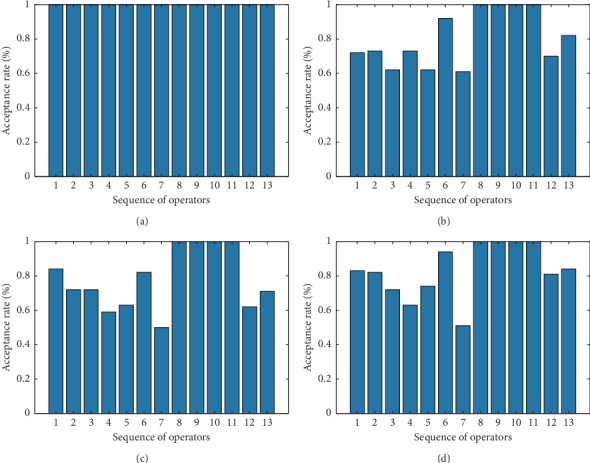
Operator acceptance rates under (a) AM, (b) GD, (c) OI, and (d) SA.

To study effects of acceptance criteria on solution results, acceptance rates of LLHs as per four acceptance criteria using selection strategy TS were statistically analysed (shown in Figure 4).

As can be found in Figure 4, according to acceptance criterion AM, all solutions are accepted, so all acceptance rates were 1; as per acceptance criteria GD and SA, good-performance LLHs (LLH_6_ and LLH_8_) had mean acceptance rates above 0.9; according to acceptance criterion OI, only improved solutions are accepted and higher acceptance rates of high-quality LLHs were achieved, but acceptance rates of ordinary-performance LLHs were lower and readily trapped in local optimum. For poor-performance LLH (LLH_7_), acceptance rates as per acceptance criteria SA and GD were 0.50 and 0.53, respectively. According to acceptance criterion SA, it is easier to reject poor-performance LLHs and accept good-performance LLHs; therefore, acceptance criterion SA has more positive impact on improvement of solutions to a biobjective LRP, which validates experimental conclusions in this paper.

### 3.5. Experimental Comparison between Algorithms MOHH and NSGA-II

To further illustrate effectiveness of MOHH algorithm in solving biobjective LRPs, TS-SA serves as an HLH strategy combination of MOHH for instance solving, as compared with algorithm NSGA-II. For the sake of fairness, NSGA-II adopts the same heuristic that MOHH adopts (Table 3), while multiobjective performance metrics were used to assess the resulting Pareto fronts (Table 4).

In nine instances, MOHH had 5.2 Pareto solutions on average, while NSGA-II obtained 4.6 Pareto solutions. In terms of performance metrics, MOHH was slightly superior to NSGA-II in NPS and mean SM of MOHH-derived Pareto solution set was 65.2, while mean SM of NSGA-II-derived Pareto solution set was 79.5; therefore, MOHH-derived Pareto solution set had smaller mean scheme spacing and more uniform distribution. MOHH-derived Pareto solution set had a mean DM of 86.5, while NSGA-II-derived Pareto solution set had a mean DM of 59.7; therefore MOHH-derived solutions are superior to NSGA-II-derived solutions in diverse metrics.

### 3.6. Practical Instances of Cold Chain Logistics

It has been validated that MOHH is effective in solving biobjective LRPs. The cold chain logistics model was solved by MOHH using selection strategy TS according to acceptance criterion SA. In cold chain logistics network programming, there are usually more than one decision objective; optimization of cost alone would fail to meet benefits of the logistics company and customers; therefore, it is essential to perform multiobjective optimization of cold chain logistics.

**Figure 5:**
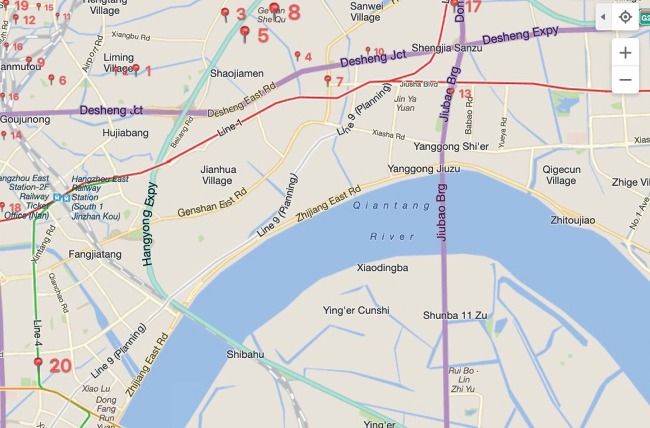
Partial distribution map of supermarket in Hangzhou.

This section focuses on the cold chain logistics of seafood of a supermarket in Hangzhou City, and the subbranches of the supermarket have a wide distribution and range of services. The distribution of subbranches (i.e., customers) is shown in Figure 5.

Aiming at improving competitiveness of cold chain logistics, the supermarket selected five distribution centres in Hangzhou. Five distribution centres met the requirements of the cold chain. In this paper, 30 subbranches are selected: 1, 2, 3,…, 30, and the distribution centres are represented by 31, 32, 33, 34, and 35. The demand and time windows are known, and the distance between each distribution centre and each subbranch is known. Each distribution centre is equipped with a dedicated refrigerated truck for distribution. The maximum capacity of each vehicle is 5 tons. The average traveling speed is 40 km/h on urban roads. The unit cooling cost *w*_2_ is 2 Yuan/Kcal. The unit penalty cost *α* of the satisfaction time window is 2 Yuan/h, and the unit penalty cost *β* is 3 Yuan/h later than the customer satisfaction time; the external temperature *T* is 77 K, and the temperature *T*_0_ in the refrigerator is 35.6 K. The thermal conductivity *R*_1_ of the compartment is 80, the thermal conductivity *R*_2_ is 0.03, the heat transfer area *S* of the refrigerated truck is 17.25 m^2^, the door area *S*_d_ is 5.75 m^2^, the transportation cost per kilometre *φ* is 4 Yuan/km, and *ρ*^*∗*^/*ρ*_0_ is 0.415/0.155 L/km; diesel price of Zhejiang province is 7.4 Yuan/L; cargo damage cost *c* is 2.0 Yuan/kg; time sensitivity coefficient *φ* is 0.005. The distribution information of distribution centres and subbranches is provided in Tables 5 and 6.

**Figure 6:**
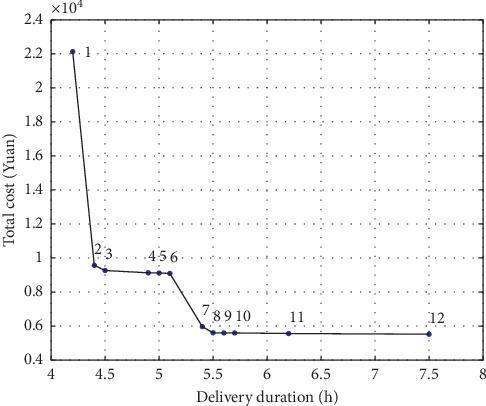
Pareto front of distribution in cold chain logistics.

Experimentally obtained Pareto solutions are shown in Figure 6. Solving biobjective cold chain logistics LRP by MOHH resulted in a total of 12 Pareto optimal solutions. Distribution time and costs corresponding to Pareto solutions were analysed in Table 7, where Cost_1_ represents path cost, Cost_2_ represents fuel cost, Cost_3_ represents refrigeration cost of the refrigerated truck, Cost_4_ represents cargo damage cost of cold chain logistics, Cost_5_ represents penalty cost of time window, and Cost_6_ represents operation cost of each distribution centre.

As described in Table 7, all the above 12 distribution schemes are Pareto optimal solutions. Pareto No. 1 corresponds to a solution with the shortest distribution time (4.2 h), but corresponding total cost reached 22126.0 Yuan; Pareto No. 12 corresponds to a solution with the minimum total cost (5522.0 Yuan), but its distribution time reached 7.5 h.

**Figure 7:**
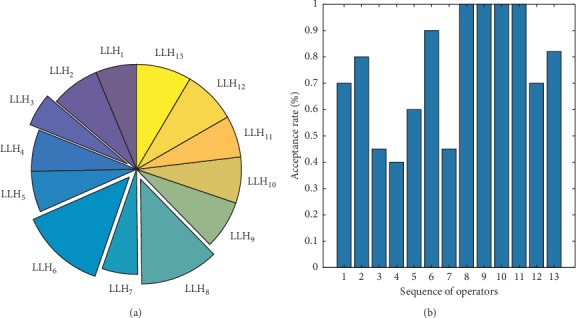
(a) Usage rate of operators. (b) Acceptance rate.

To validate the effect of MOHH in solving instances, LLH utilization during algorithm iteration was statistically analysed, as shown in Figure 7. Based on Figure 7, in selection strategy TS, utilization rates of LLH_6_ and LLH_8_ were the highest (13.1% and 12.1%, respectively), while utilization rates of LLH_3_ and LLH_7_ were only 5.1% and 5.5%, respectively. Moreover, according to acceptance criterion SA, acceptance rate of LLH_6_ was more than 0.9, while acceptance rates of LLH_3_ and LLH_7_ were 0.53 and 0.52, respectively. According to statistical analysis in Section 3.3, LLH_6_ and LLH_8_ had the best optimization effect, while LLH_7_ had worse performance. In process of solving cold chain logistics instances, in MOHH, LLH_6_ and LLH_8_ were still utilized and accepted at higher rates, while LLH_7_ was utilized and accepted at lower rates; it can therefore be concluded that MOHH has better utilization of good-performance LLHs and better improvement of solution quality.

Practicality of MOHH was validated by instance analysis. For multiobjective LRPs, difference in objective preference will result in difference in distribution scheme and great differences in distribution centre selection and routing. Nonetheless, multiobjective optimization is capable of balancing between the logistics company and customer demand, and to meet different demands, different distribution schemes can be used to enable balance of interests between the logistics company and customers where possible. Furthermore, with total cost and distribution time as study objectives, it is in favour of decision maker of the logistics company in offering diverse path options.

## 4. Conclusions

In this paper, one biobjective mathematical model is established for cold chain logistics distribution system and is used to improve economic benefit, environmental benefit, timeliness, and customer satisfaction of the logistics distribution system. The first primary objective of the established multiobjective model is to optimize logistics distribution cost, consisting of path cost, fuel cost, refrigeration cost of the refrigerated truck, cargo damage cost of cold chain logistics, penalty cost of time window, and operation cost of distribution centres, while the second primary objective is to optimize timeliness of the distribution system or distribution time. To address this problem, MOHH algorithm was designed for model solving. As to HLH strategy for MOHH, the best HLH strategy combination was obtained from combinations of four selection strategies and four acceptance criteria.

Based on experimental results, TS-SA was the best HLH strategy. Further comparison with solution results of classic multiobjective solving algorithm NSGA-II validated effectiveness of MOHH, and, finally, solving instances of cold chain logistics by MOHH validated practicality of the algorithm, while the resulting Pareto solution set offers diverse options for a decision maker to select an appropriate distribution scheme depending on actual demand.

The next study will focus on considering the use of more actual constraints in cold chain logistics such as heterogeneous vehicle [29] and simultaneous pickup and delivery [30], so that the problem can be more realistic. Higher-performance HLH strategy will be designed to improve solution quality and stability of the currently proposed MOHH. Moreover, we will focus on the alternative frameworks of MOHH like [31, 32] and the framework that has a digital twin to repatriate at runtime [33]. In addition, consideration of other carbon emission models will become one of the focuses in the next work.

## Figures and Tables

**Table 1:** Experimental results of HLH strategies in solving biobjective LRP.

Selection strategy	Acceptance criteria	NPS	Distribution time		Total cost	
			Min.	Max.	Min.	Max.
AS	AM	4	1152.2	1256.2	13978.5	16646.6
	GD	7	1103.7	1444.9	14524.0	16105.5
	OI	5	1045.2	1138.8	14419.8	14849.3
	SA	5	1058.4	1131.2	14234.3	16076.0

CF	AM	4	1085.3	1124.2	14748.2	16377.2
	GD	5	1072.6	1225.7	14975.8	16379.2
	OI	4	1068.9	1206.7	15217.9	16170.7
	SA	6	1113.3	1149.2	14941.1	16424.2

SR	AM	4	1058.2	1109.2	13857.2	15345.4
	GD	3	1276.4	1323.0	17630.1	19780.0
	OI	3	1041.8	1183.0	13479.0	14590.0
	SA	5	1061.0	1211.8	14588.0	15708.7

TS	AM	6	1056.3	1186.6	14147.4	14642.1
	GD	5	1145.0	1215.0	14800.5	17415.9
	OI	6	1222.2	1437.5	16453.1	19246.3
	SA	7	1042.6	1172.3	13316.7	13316.7

**Table 2:** Comparison of performance metrics of different HLH strategies for MOHH.

Selection strategy	Acceptance criteria	NPS	SM	DM
AS	AM	4	147.3	97.8
	GD	7	206.7	101.0
	OI	5	134.7	46.8
	SA	5	155.6	90.2

CF	AM	4	629.4	78.6
	GD	5	393.0	80.6
	OI	4	292.1	58.8
	SA	6	303.0	84.0

SR	AM	4	424.8	81.5
	GD	3	315.9	76.5
	OI	3	29.1	56.8
	SA	5	269.6	71.7

TS	AM	6	58.9	57.0
	GD	5	134.1	105.3
	OI	5	217.9	114.9
	SA	7	26.3	101.1

**Table 3:** Comparison between algorithms MOHH and NSGA-II in solution results.

Instance	Solution algorithm	NPS	Distribution time		Total cost	
			Min.	Max.	Min.	Max.
R101	MOHH	6	1222.1	1374.3	16523.0	18479.0
	NSGA-II	5	2045.7	2343.5	17410.5	18215.5

R102	MOHH	6	1193.8	1339.0	16525.1	18208.2
	NSGA-II	6	2113.9	2222.7	16868.0	17499.2

R103	MOHH	5	1179.2	1336.0	16310.7	18164.6
	NSGA-II	4	2173.3	2241.7	16920.8	18929.3

R104	MOHH	5	1166.0	1262.8	16108.6	18826.3
	NSGA-II	3	2127.3	2149.9	19552.7	19915.6

R105	MOHH	5	1157.3	1301.6	16927.0	17444.0
	NSGA-II	4	2137.2	2222.7	17760.5	18476.2

R106	MOHH	6	1207.8	1288.9	15714.2	16235.3
	NSGA-II	5	2248.8	2603.4	17007.0	17393.8

R107	MOHH	6	1178.8	1312.7	15500.9	17250.4
	NSGA-II	5	2106.4	2134.6	15368.0	16041.6

R108	MOHH	3	1135.0	1184.1	13818.2	15053.1
	NSGA-II	5	2135.2	2472.4	15600.0	16038.5

R109	MOHH	5	1229.4	1406.0	16358.5	18889.2
	NSGA-II	4	2053.3	2383.1	15302.5	15932.8

**Table 4:** Comparison between MOHH and NSGA-II in performance metrics.

Instance	Solution algorithm	NPS	SM	DM
R101	MOHH	6	53.8	101.1
	NSGA-II	5	56.9	77.2

R102	MOHH	6	94.3	90.9
	NSGA-II	6	79.2	60.0

R103	MOHH	5	22.7	94.9
	NSGA-II	4	76.3	89.6

R104	MOHH	5	60.3	110.6
	NSGA-II	3	81.2	32.2

R105	MOHH	5	49.0	52.2
	NSGA-II	4	31.3	53.8

R106	MOHH	6	54.9	63.5
	NSGA-II	5	162.4	52.3

R107	MOHH	6	107.5	97.8
	NSGA-II	5	111.8	52.9

R108	MOHH	3	70.9	61.8
	NSGA-II	5	83.5	60.2

R109	MOHH	5	73.2	106.0
	NSGA-II	4	32.5	59.2

**Table 5:** Information of distribution centres.

No.	Location	Capacity	Fixed cost
31	(120.213287, 30.316118)	50	4.0
32	(120.108078, 30.254743)	40	3.5
33	(120.217886, 30.172352)	70	7.0
34	(120.177067, 30.243263)	55	4.0
35	(120.160395, 30.311877)	60	5.5

**Table 6:** Information of subbranches.

No.	Location	Demand	Service time	Time windows
1	(120.217936, 30.296719)	0.8	0.3	[0.0, 2.0]
2	(120.201977, 30.263709)	1.1	0.3	[1.0, 2.0]
3	(120.123652, 30.290270)	0.8	0.3	[0.0, 2.0]
4	(120.217219, 30.206380)	1.3	0.3	[3.0, 5.0]
5	(120.190461, 30.341832)	1.1	0.3	[3.0, 5.0]
6	(120.169804, 30.282714)	1.3	0.3	[3.0, 6.0]
7	(120.046035, 30.263151)	0.6	0.3	[2.0, 5.0]
8	(120.195807, 30.185674)	0.9	0.3	[3.0, 6.0]
9	(120.234088, 30.197576)	0.5	0.3	[4.0, 6.0]
10	(120.272501, 30.334870)	1.3	0.3	[2.0, 4.0]
11	(120.125605, 30.313614)	1.3	0.3	[3.0, 6.0]
12	(120.102611, 30.300304)	0.9	0.3	[2.0, 4.0]
13	(120.284870, 30.150627)	1.4	0.3	[1.0, 4.0]
14	(120.182657, 30.264054)	1.2	0.3	[0.0, 2.0]
15	(120.166853, 30.269039)	0.5	0.3	[2.0, 4.0]
16	(120.207953, 30.249617)	1.2	0.3	[3.0, 5.0]
17	(120.282055, 30.169434)	1.1	0.3	[1.0, 3.0]
18	(120.156923, 30.280374)	0.7	0.3	[3.0, 5.0]
19	(120.174242, 30.196310)	1.2	0.3	[0.0, 2.0]
20	(120.176285, 30.219921)	1.9	0.3	[2.0, 6.0]
21	(120.093142, 30.340724)	1.5	0.3	[3.0, 5.0]
22	(120.232744, 30.209315)	1.6	0.3	[0.5, 2.0]
23	(120.178319, 30.283681)	0.8	0.3	[1.0, 2.0]
24	(120.201109, 30.208160)	0.6	0.3	[1.0, 3.0]
25	(120.191338, 30.355956)	1.6	0.3	[3.0, 6.0]
26	(120.180049, 30.252984)	1.4	0.3	[0.5, 2.0]
27	(120.166303, 30.309546)	0.5	0.3	[1.0, 3.0]
28	(120.238472, 30.358900)	0.8	0.3	[3.0, 6.0]
29	(120.177225, 30.249727)	1.1	0.3	[0.5, 2.0]
30	(120.220017, 30.315313)	1.4	0.3	[1.0, 3.0]

**Table 7:** Analysis of cost versus distribution time.

No.	Delivery time (h)	Cost_1_	Cost_2_	Cost_3_	Cost_4_	Cost_5_	Cost_6_	Total cost (Yuan)
1	4.2	304.0	516.9	624.3	109.2	71.6	20500	22126.0
2	4.4	288.3	515.2	642.1	119.9	69.2	8000	9565.5
3	4.5	253.1	412.6	446.2	79.5	73.4	8000	9264.8
4	4.9	302.9	541.1	592.1	125.9	65.9	7500	9127.9
5	5.0	308.0	542.0	579.7	122.5	64.6	7500	9116.8
6	5.1	281.2	518.0	605.0	126.8	61.8	7500	9092.8
7	5.4	350.2	635.2	770.1	151.7	60.3	4000	5967.5
8	5.5	281.3	510.5	625.7	122.0	64.7	4000	5604.2
9	5.6	273.3	520.5	602.7	134.3	65.8	4000	5596.6
10	5.7	279.0	507.7	616.3	121.9	69.4	4000	5594.3
11	6.2	278.5	501.5	599.1	118.4	63.7	4000	5561.2
12	7.5	275.7	499.1	565.7	118.8	62.7	4000	5522.0

## Data Availability

The data used to support the findings of this study are available from the corresponding author upon request.
